# The Landscape of A-to-I RNA Editome Is Shaped by Both Positive and Purifying Selection

**DOI:** 10.1371/journal.pgen.1006191

**Published:** 2016-07-28

**Authors:** Yao Yu, Hongxia Zhou, Yimeng Kong, Bohu Pan, Longxian Chen, Hongbing Wang, Pei Hao, Xuan Li

**Affiliations:** 1 Key Laboratory of Synthetic Biology, CAS Center for Excellence in Molecular Plant Sciences, Institute of Plant Physiology and Ecology, Shanghai Institutes for Biological Sciences, Chinese Academy of Sciences, Shanghai, China; 2 Department of Physiology, Michigan State University, East Lansing, Michigan, United States of America; 3 Key Laboratory of Molecular Virology and Immunology, Institute Pasteur of Shanghai, Chinese Academy of Sciences, Shanghai, China; 4 Shanghai Center for Bioinformation Technology, Shanghai Industrial Technology Institute, Shanghai, China; Aarhus University, DENMARK

## Abstract

The hydrolytic deamination of adenosine to inosine (A-to-I editing) in precursor mRNA induces variable gene products at the post-transcription level. How and to what extent A-to-I RNA editing diversifies transcriptome is not fully characterized in the evolution, and very little is known about the selective constraints that drive the evolution of RNA editing events. Here we present a study on A-to-I RNA editing, by generating a global profile of A-to-I editing for a phylogeny of seven *Drosophila* species, a model system spanning an evolutionary timeframe of approximately 45 million years. Of totally 9281 editing events identified, 5150 (55.5%) are located in the coding sequences (CDS) of 2734 genes. Phylogenetic analysis places these genes into 1,526 homologous families, about 5% of total gene families in the fly lineages. Based on conservation of the editing sites, the editing events in CDS are categorized into three distinct types, representing events on singleton genes (type I), and events not conserved (type II) or conserved (type III) within multi-gene families. While both type I and II events are subject to purifying selection, notably type III events are positively selected, and highly enriched in the components and functions of the nervous system. The tissue profiles are documented for three editing types, and their critical roles are further implicated by their shifting patterns during holometabolous development and in post-mating response. In conclusion, three A-to-I RNA editing types are found to have distinct evolutionary dynamics. It appears that nervous system functions are mainly tested to determine if an A-to-I editing is beneficial for an organism. The coding plasticity enabled by A-to-I editing creates a new class of binary variations, which is a superior alternative to maintain heterozygosity of expressed genes in a diploid mating system.

## Author Summary

One prevalent form of RNA editing is the deamination of adenosines (A-to-I editing) in the precursor mRNA molecules, pertaining to most organisms in the metazoan lineage. While examples of A-to-I editing on critical genes have been known for years, it has not been fully characterized how A-to-I editing shapes the transcriptome and proteome in the evolution. To understand how A-to-I editing affects genes’ evolution and how itself is constrained by selection, we generated a global profile of A-to-I editing for a phylogeny of seven fly species, a model system representing an evolutionary timeframe of about 45 million years. We are focused on 5150 editing sites (of totally 9281 identified) located in the coding region of 2734 genes. Our analysis revealed the evolution dynamics of A-to-I editing sites and functional specificity of targeted genes. The shifting patterns of A-to-I editing are documented during holometabolous development and in post-mating response in flies. This work points to the important roles of regulated RNA editing in animal development and offers new insight into the evolution of A-to-I editing events and their harboring genes.

## Introduction

Since it was first discovered over 20 years ago [1] RNA editing has emerged as an important source of genetic coding variations in diverse life forms. One prominent mechanism for RNA editing is the deamination of adenosines in the precursor mRNA molecules, pertaining to most organisms in the metazoan lineage, including insects and mammals [2–4]. The deamination event, namely A-to-I editing, converts specific adenosines (A) to inosines (I). Inosines are decoded as guanosines (G) in translation, thus resulting in codon changes that often lead to amino acid substitutions in the protein products. In addition to genetic recoding, A-to-I editing is also known to affect alternative splicing [5,6], modify microRNAs, and alter microRNA target sites [5,7,8]. The major component of the A-to-I RNA editing machinery is the so called adenosine deaminases acting on RNA (ADAR) family of enzymes, which act on double stranded RNA structures (dsRNAs) within the substrate molecules [3,4,9]. Details about substrate targeting and regulation of editing activities are sparse; however, evidence indicates that A-to-I editing was cotranscriptional [10], and the ADAR targeting sites were delineated to prefer certain non-random sequence patterns [11,12], and depended in large part on the tertiary structure of RNA duplexes [4,13,14].

Genetic variability generated by A-to-I RNA editing expands the diversity and complexity of transcriptome, which serves as an important mechanism helping support critical biological functions. Lacking A-to-I RNA editing due to *ADAR* mutation in animal models resulted in embryonic or postnatal lethality in mice [15,16], or displaying neurological defects in flies [17,18]. Many A-to-I editing targeted genes were documented in previous studies in human, mice, rhesus, and fly [19–22]. Reported cases of editing targets include the neuronal receptors [23,24], ion transporters [25], and immune response receptors [26]. While examples of A-to-I RNA editing on critical genes have been known for years, from the evolutionary perspective how and to what extent that A-to-I editing diversifies and shapes the transcriptome and proteome is not fully characterized in the evolution. And very little is known about how RNA editing itself is constrained by selective forces through evolution. There are variable views on the adaptive potentials provided by A-to-I RNA editing. While it was suggested that A-to-I editing on coding genes was non-adaptive from the studies on rhesus and human [22,27], the ‘continuous probing’ hypothesis presented some likely scenario for ‘functional significant editing sites’ [28]. This hypothesis proposed that novel RNA editing sites that emerged on transient double-strand RNA structures, were continuously probed during evolution and became the basis for adaptive selection. And more recently, the non-synonymous high-level A-to-I editing events were proposed to be beneficial in human [29].

The next-generation sequencing technology and the Model Organism ENCyclopedia Of DNA Elements (modENCODE) Project [30] enabled an unprecedented resource on the model organisms, like *Drosophila* and *Caenorhabditis*, that made it possible for the multi-genome large scale analysis to compare RNA editing patterns in the evolution. To explore the landscape of RNA editing and characterize the selective constraints imposed on A-to-I editing through evolution, we assembled a study based on the modENCODE resource, involving seven *Drosophila* species for which there were both reference genome and corresponding transcriptome sequencing data available. The study was also complemented with data from other sources, including NCBI Sequence Read Archive (SRA) [31], NCBI Gene Expression Omnibus (GEO) [32], FlyBase [33], and FlySNPdb database [34]. Using the *Drosophila* genus as a model system that represents an evolutionary timeframe of approximately 45 million years, we identified a total of 9281 A-to-I RNA editing events. Validations of the events were performed by comparing with results of previous studies and with data from fly tissue/development samples or *ADAR* mutants, and by carrying out mass array-based validation experiments. Through phylogenetic analysis, the A-to-I RNA editing events were categorized into three distinct types based on the conservation of the editing sites. The profiles and physiological significance of each editing type were analyzed in association with selective constraints through evolution and with functional enrichment in the context of gene ontology (GO). Further evidence revealed the changing patterns of different editing types during holometabolous development and in post-mating response, thus implying the active involvement of RNA editing in short-term response and in normal physiological processes. This work represents a comprehensive study on A-to-I RNA editing in flies at an unprecedented scale, which offers new insight into the evolutionary dynamics of A-to-I editing events, and the critical roles of RNA editing events in fly nervous system.

## Results

### Generating a reference set of A-to-I RNA-editing events in *Drosophila*

**Fig 1:**
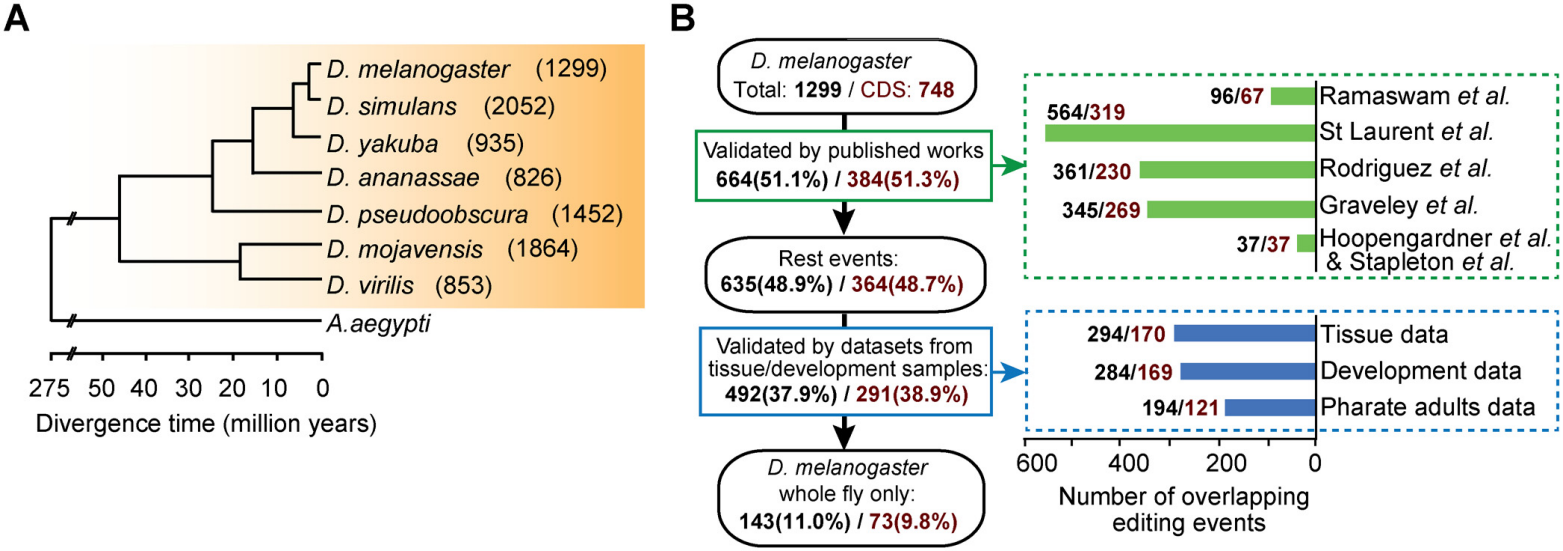
Generating the reference set of A-to-I RNA editing events in closely related fly species. (A) The evolutionary tree of the seven *Drosophila* species. The branching order and the divergence times were derived from the TimeTree database [35]. The bracketed numbers to the right indicate the editing events identified for each species. (B) Validation of the *D*. *melanogaster* subset of A-to-I editing events. 664 events were first mapped to the published lists (S5 Table). For the rest 635, 492 were validated by the tissue/development data sets (S5 Table). Those supported by the published lists [10,21,36–39] and by the tissue/development data sets (S2, S3 and S6 Tables) were broken down to original sources, represented by horizontal bars (green and blue) to the right.

To explore the A-to-I RNA editome and characterize the evolutionary dynamics of the RNA editing events, we first sought to compile a reference set of events from evolutionarily related model organisms. The *Drosophila* genus offers some unique advantage for our purpose, as the flies originate from a common ancestor from approximately 45 million years ago (mya) (Fig 1A), and many have well annotated quality genome and corresponding transcriptome sequencing data. Upon careful searching the modENCODE data collections, the seven fly species, *D*. *ananassae*, *D*. *melanogaster*, *D*. *mojavensis*, *D*. *pseudoobscura*, *D*. *simulans*, *D*. *virilis*, *D*. *yakuba*, were found to meet our needs. Our study utilized the genome and transcriptome sequencing data from samples of whole fly, different tissue types and developmental stages (S1–S3 Tables; see Methods for details). Additional data were acquired to complement the modENCODE data, including *D*. *melanogaster* pharate adult dataset, *D*. *pseudoobscura* and *D*. *simulans* tissue datasets, *D*. *melanogaster* genome re-sequencing data, and head RNA-Seq data of the *Adar*^*5G1*^ mutant and paired wild type strain *w*^*1118*^ (S6 and S12 Tables; see Methods for details).

To identify A-to-I RNA editing events for the seven species, their whole fly deep-sequencing transcriptome data (S1 Table) were initially analyzed. To call A-to-I RNA editing events, we used a modified pipeline (see Methods for details) similar to what was described by Ramaswami [36]. We identified totally 9281 A-to-I editing candidate events to generate a reference set, ranging from 826 in *D*. *ananassae* to 2052 in *D*. *simulans* (Fig 1A and S4 Table). When compared to non A-to-G mismatches from our pipeline, percentage wise the A-to-G editing change was 16- and 14-fold higher than the average of other base change types in all sites and in CDS sites, respectively (S1 Fig). Assuming that all non-canonical mismatches were background noise, and the error rates for all 12 base change were equal, the false positive rate for A-to-G change type was estimated to be 5.59% for all sites, and 6.32% for CDS sites [36]. (Annotation of A-to-I editing events is described in the next section.) These values were in line with those of previous studies, which suggested that almost all non-canonical base changes were due to sequencing errors or alignment artifacts [20,36]. To validate the A-to-I editing events and estimate the error rate from our process, we sampled and scrutinized the subset from *D*. *melanogaster*, which included 1299 events. First, we compared the *D*. *melanogaster* subset with those from previous studies on the same species. 37, 345, 361, 96, and 564 A-to-I editing events from the studies of Hoopengardner and Stapleton [37,38], Graveley [39], Rodriguez [10], Ramaswami [36] and St Laurent [21] overlapped with ours, respectively (Fig 1B and S5 Table). Notably, 37 of the 44 events collected and manually validated by Hoopengardner and by Stapleton were included in our *D*. *melanogaster* subset. Collectively, the combined data from those previous studies covered 664 (51.1%) of the editing events in our *D*. *melanogaster* subset. Second, to further examine the rest 635 events not overlapping with previous studies, we obtained additional transcriptome sequencing data sets generated from pharate adults (S6 Table) [40], from nine tissue types (S2 Table) [41], and from four developmental stages (S3 Table). Within the 635 events, 194, 294, and 293 were found with the above datasets, respectively (Fig 1B and S5 Table). Merging together they supported 492 bona fide A-to-I editing events in the group of 635, which account for another 37.9% of the *D*. *melanogaster* subset. Taken together, 1156 of 1299 events (89.0% of the *D*. *melanogaster* subset) either overlapped with the previous studies or were reproduced with new tissue/development samples. When counting editing events in gene coding regions (CDS) separately, 675 of 748 CDS events (90.2%) were supported by previous data (Fig 1B), which is slightly higher than that for all events.

Third, to validate the identified editing events catalyzed by the ADAR enzyme, we obtained and analyzed the RNA-Seq datasets from paired *D*. *melanogaster* samples of wild-type strain (*w*^*1118*^) and *Adar*^*5G1*^ mutant [36]. The *Adar*^*5G1*^ mutant flies were found previously to be defective in A-to-I RNA editing [36]. Out of 1299 events in the *D*. *melanogaster* subset, 523 were present in the head of the wild type. However, in the head of the *Adar*^*5G1*^ mutant, 485 of the 523 (92.7%) were found to have adenosine residues only (S7 Table), confirming the vast majority of identified events are associated with ADAR activity in *D*. *melanogaster*. The false positive rate estimated with the *Adar*^*5G1*^ mutant data is 7.3% (38/523) for all events and 8.7% (27/312) for CDS events, in line with other studies using similar scheme [10,36].

Forth, we also estimated the false positive rate in the *D*. *melanogaster* subset that is due to possible genomic variation, e.g. single nucleotide polymorphism (SNP). We first created a genomic variant database for *D*. *melanogaster*, combining the SNP data from FLYSNPdb [34] with variants identified from genome sequencing data (see Methods for details). We then crosschecked our *D*. *melanogaster* subset with the genomic variant database (S1 Text). We reasoned that if an A-to-I editing site was found to match an A/G genomic variant, the editing event might be a suspect, possibly resulted from a genomic variant. 110 of the 1299 (8.95%) editing events in the *D*. *melanogaster* subset and 74 of 748 (9.89%) CDS events found A/G correspondents in our genomic variant database. So the estimated false positive rate due to genomic variation is 8.95% for all editing events (9.89% for CDS events) by our pipeline.

We attempted similar analysis to estimate the success rate of A-to-I editing events in other fly species. We were able to recover 74.24% and 75.91% of all events (72.77% and 70.36% of CDS events) (S14 Table) only for two species, *D*. *pseudoobscura* and *D*. *mojavensis*, respectively, with RNA-seq data from separate sources (S12 Table). Due to limited tissue types and smaller datasets from these species, the recovery rates for *D*. *pseudoobscura* and *D*. *mojavensis* are lower than that (86.0%) for *D*. *melanogaster*. Finally, we carried out mass array-based validation experiments using the Sequenom's MassARRAY platform as described [20,22]. On randomly selected A-to-I editing events form all seven fly species, the overall success rates were 86.7% for all events, and 89.9% for CDS events. So using mass array-based validation approach, the non-confirming rates for all seven species were 13.3% for all events and 10.1% for CDS events, respectively. They are likely to represent the upper limit of the false positive rate in our work, as many events in the non-confirming category may be missed due to the lower sensitivity of mass array genotyping compared to RNA-seq [20]. Looking more closely into species, the success rates estimated for *D*. *melanogaster*, *D*.*mojavensis*, *D*. *simulans*, *D*. *pseudoobscura*, *D*. *yakuba*, *D*. *ananassae*, and *D*. *virilis* were 84.6%, 88.5%, 100.0%, 71.0%, 92.6%, 90.5%, and 83.3%, respectively, for all events, and 82.4%, 94.4%, 100.0%, 91.3%, 91.3%, 92.9%, and 76.5%, respectively, for CDS events (S16 Table).

In summary, analyses of the sampled data suggest our process is effective and reliable for the identification of A-to-I editing events in *Drosophila*. The seven fly species were found to have comparable success/false positive rates when estimated using mass array-base validation approach. These results are in line with those of the previous studies [21,36], re-enforcing confidence in our analysis pipeline.

### Global profile of A-to-I RNA editing events in *Drosophila*

To characterize the genome distribution of A-to-I RNA editing events in *Drosophila*, the editing sites were to be annotated with the gene structure information from FlyBase. However, in the current genome releases, the gene models for *D*. *yakuba*, *D*. *ananassae*, *D*. *simulans*, *D*. *mojavensis*, *and D*. *virili* lacked the definition for 5’- and 3’-UTRs (untranslated regions). So we first redefined the UTR boundaries for gene models in these five species with the help of trancriptome sequencing data (see Methods for details). The UTRs for a total of 62,193 gene models were completed (S2 Text). The A-to-I editing sites were then annotated with the newly updated gene structures (Table 1 and S4 Table). Between 16.8% and 32.1% of events were found in the intronic or intergenic regions in various species (Table 1). Some events in intergenic regions coincided with non-coding RNAs. For example, in *D*. *malenogaster* 30 events were located within its non-coding RNA sequences (S8 Table). The exonic events (in UTRs or CDS) accounted for 74.5% of all events, for which the majorities (74.5%) were found in the CDS that could lead to amino acid coding changes. Indeed, with the exceptions of *D*. *virilis* and *D*. *ananassae*, A-to-I editing events in CDS regions occupied more than 50% of all events. The RNA editing events were significantly biased toward CDS regions (S17 Table, *Fisher's Exact Test*, *p-value* < 5.24E-60), strongly implying function of RNA editing on gene coding sequences in flies.

**Table 1:** Annotation of A-to-I RNA editing events according to the gene models of each species.

Species	Distributions					Total
	CDS	Intronic	UTR5	UTR3	Intergenic	
*D*. *simulans*	**1212**	141	80	308	311	2052
*D*. *melanogaster*	**748**	100	51	233	167(30) *	1299
*D*. *yakuba*	**481**	70	34	170	180	935
*D*. *ananassae*	**396**	126	16	134	154	826
*D*. *pseudoobscura*	**918**	67	45	212	210	1452
*D*. *mojavensis*	**1066**	181	44	252	321	1864
*D*. *virilis*	**329**	137	39	143	205	853

*Number in parenthesis represents sites located in non-coding RNA sequence.

**Fig 2:**
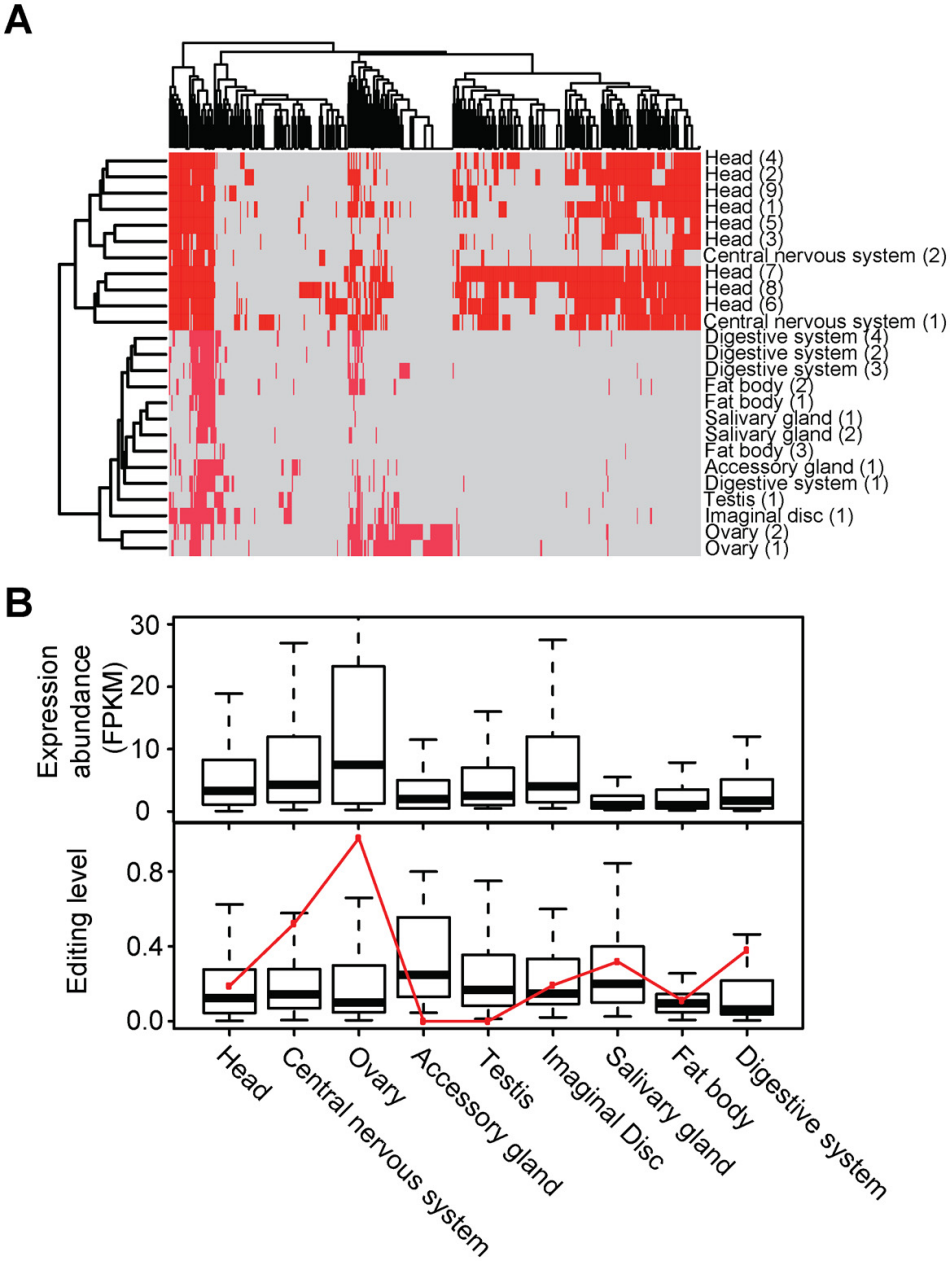
The profiles of A-to-I RNA editing in *D*. *melanogaster*. (A) Hierarchical clustering of detected A-to-I editing events across samples of nine tissue types, including head, central nervous system, ovary, accessory gland, testis, imaginal disc, salivary gland, fat body, and digestive system (see S2 Table for sample details). The dendrogram on the top illustrates the classification among A-to-I editing events, and to the left the grouping of tissue samples. (B) Tissue profile of A-to-I editing events. The expression of edited genes (top panel) and the editing level of events (bottom panel) are shown in box plots. The gene expression is measured using FPKM reported by Cufflinks (v2.1.1) [45]. The editing level is defined by the percentage of edited reads in total reads covering an editing site. The expression level of *ADAR* gene is indicated by a red line (bottom panel), which is normalized to a scale of 0 to 1 (with the expression level in ovary being 1).

To reveal the tissue profile of A-to-I RNA editing events, we performed hierarchical clustering analysis on the *D*. *melanogaster* subset cross nine tissue types (Fig 2A). The A-to-I editing events grouped tissue samples into two apparent clusters, namely nervous tissues (central nervous system, and head) versus the rest (accessory gland, fat body, ovary, salivary gland, digestive system, imaginal disc, and testis). We next analyzed the profiles of genes targeted by RNA editing in the *D*. *melanogaster* tissues. Considerable variances were displayed in both gene expression abundance and editing level across the tissue types (Fig 2B). To determine the effect of *ADAR* gene [3,42] expression on RNA editing level in flies, we plotted *ADAR* expression level in all the tissues (Fig 2B, bottom panel). While *ADAR* exhibited a large variation cross tissue types, to our surprise a poor correlation between *ADAR* expression and median A-to-I editing levels in *D*. *melanogaster* tissues was observed (Kendall’s tau-b coefficient = -0.315). Other confounding factors apart from *ADAR* expression are suspected to be involved in the regulation of A-to-I editing activity in tissues. Representing the first documented profile for A-to-I editing in flies, the large variances in editing levels in tissues resemble those found in mice [20], rhesus [22], or human [43,44].

Secondary structure forming around the RNA editing sites plays important role in the substrate-enzyme recognition, thus affecting the efficiency of A-to-I RNA editing. Structural RNAs have lower folding energy [46–49]. We calculated the minimum free energy for secondary structures [50] for the identified editing sites in *D*. *melanogaster* and compared them with those for randomly picked sites. Significant difference was observed between sequences flanking editing sites and those random ones (S4 Fig, *Wilcoxon-Mann-Whitney rank sum test*, *p-value* = 5.094E-06). The lower median minimum free energy from the editing sites indicates a tendency to form more stable secondary structure around them. In comparison, early studies [11–14] suggested that both the secondary structure and the sequencing context of editing sites were important factors affecting the editing activities. However, apart from the lower median minimum free energy, no strict sequence feature concerning the RNA editing sites was identified in our work.

### Three distinct types of A-to-I RNA editing events in CDS regions

The large fraction of A-to-I editing events concentrating in the CDS regions in *Drosophila* has a strong functional implication of RNA editing on coding genes. It is imperative to ask what adaptive advantage in evolution, if any, is gained from A-to-I RNA editing.

#### Emergence of three distinct A-to-I RNA editing types

**Fig 3:**
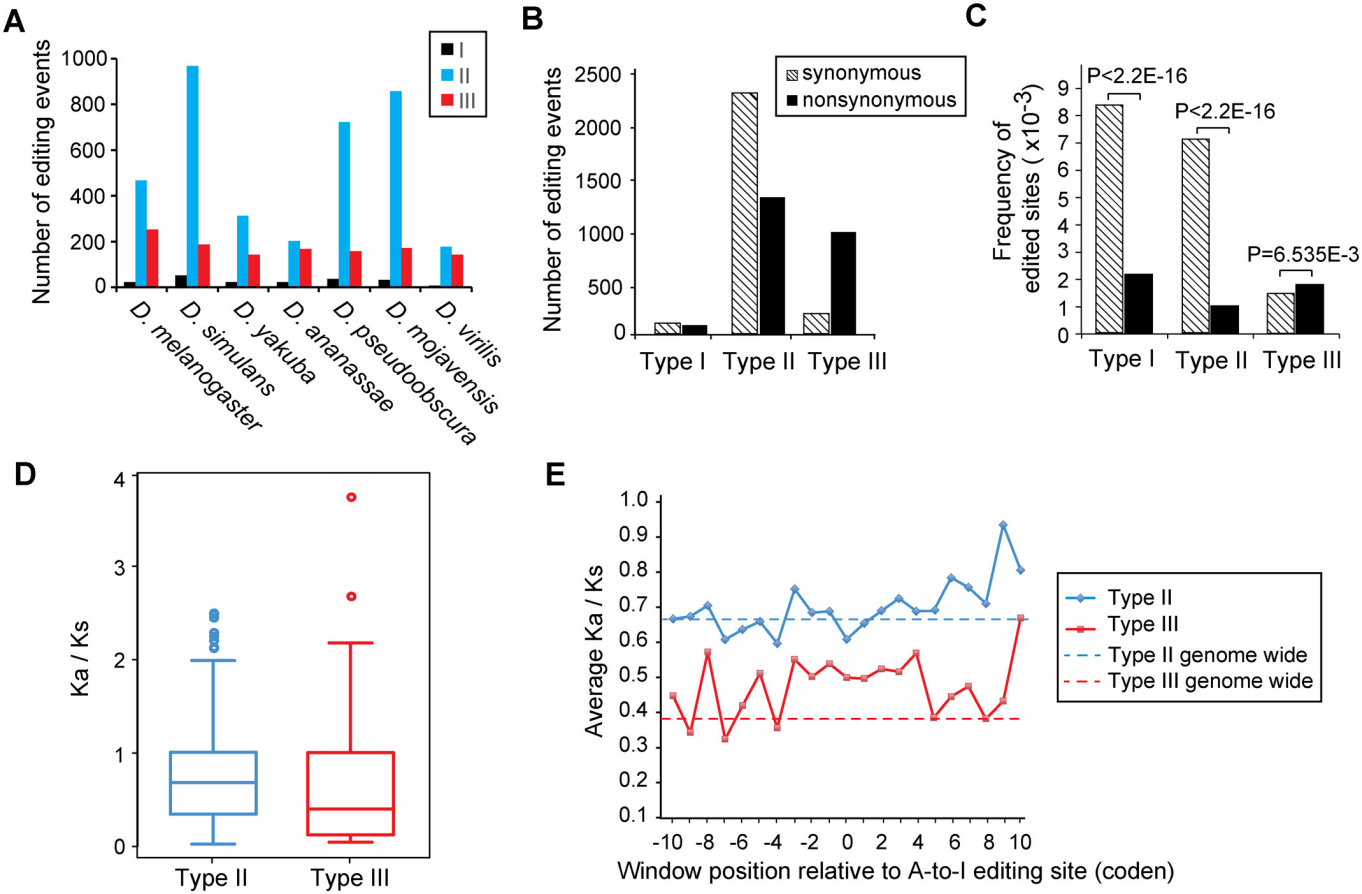
Characterization of three types of A-to-I RNA editing events in gene coding regions. (A) Number of events for A-to-I editing types in each species (see Methods for details). (B) Numbers of synonymous and non-synonymous editing events for each editing type. (C) The frequency of synonymous and non-synonymous A-to-I editing events for each editing type. The frequency is defined as the ratio of detected A-to-I synonymous (or non-synonymous) editing events over all possible A-to-I synonymous (or non-synonymous) changes in the edited genes. (D) The ratio of non-synonymous substitutions rate (*Ka*) to synonymous substitutions rate (*Ks*) shown in box plots (see Methods for details). Computation of *Ka*/*K*s values is not applicable to type I genes that are singletons by definition. (E) Average *Ka*/*Ks* values for regions near A-to-I editing sites.

We first established the phylogenetic relationship among the coding genes targeted by A-to-I RNA editing (see Methods for details). Of the total 30,434 gene families from the seven *Drosophila* species, 1526 (5.0%) (S9 Table) were found to contain 2734 genes with CDS regions harboring A-to-I editing events. The small fraction of genes being edited agrees with previous works in *D*. *melanogaster* [21,36], but is larger than that in mice or human [20,36,51]. When we looked closer at the editing events in members of the same gene families, three distinct types of events emerged based on the conservation of editing sites and their host genes. The type I events contained 206 sites found in 133 singleton genes that did not have detectable homologous gene in other fly species. The type II events contained 3716 sites found in 1393 multi-member gene families, but each occurred in one member and had no conserved event in other members of the same family. The type IIIs comprised 1231 sites found in 209 multi-member gene families, where conserved events occurred in at least two members of the same family. The type I, II, and III events occupied 4.0%, 72.1%, and 23.9% of those in CDS regions, respectively. Linking the event types back to their host species (Fig 3A), type II events were found to remain the largest fraction followed by the type IIIs and Is in each species. We reconstructed the ancestral states for type III events with GLOOME [52], which used stochastic mapping [53] to detect the gains and losses of editing events along the phylogeny. The results indicated that the majority of ‘conserved events’ were maintained even though they underwent gain and loss process in evolutionary history (S2 Fig). The analysis, for the first time, revealed new and important details about the evolution of A-to-I editing events.

#### Synonymous versus non-synonymous recoding

Next we analyzed and compared synonymous and non-synonymous amino acid changes caused by A-to-I RNA editing in each event type. The type I events produced almost equal numbers of synonymous and non-synonymous code changes (Fig 3B). However, the types II events had more synonymous code changes than non-synonymous ones, whereas the type IIIs had more non-synonymous than synonymous changes. To characterize the selective pressure the events are subject to, one has to place the editing sites in the global sequence contexts of all edited genes [22,27,54]. For the type I and II events, there were 94 and 1372 non-synonymous changes, and 112 and 2344 synonymous ones, respectively. But if all the ‘A’ residues in type I and II genes’ coding regions were edited to ‘I’, there would be 46078 and 12161184 non-synonymous changes, and 13595 and 326396 synonymous ones, respectively. The frequencies for non-synonymous editing, 2.04E-3 (94/46078) for type I and 1.09E-4 (1372/12161184) for type II, are both smaller than those for synonymous editing, 8.24E-3 (112/13594) for type I and 7.18E-3 (2344/326396) for type II, respectively. The reduction in frequency for non-synonymous editing in either type I or II events is statistically significant (*Chi-square test*, *p-value* <2.2E-16 for either type) (Fig 3C), suggesting non-synonymous editing events in both type I and II genes are deleterious and purged by purifying selection through evolution.

For the type III events, there were 1029 non-synonymous changes versus 202 synonymous ones. Again, if all ‘A’ sites in coding regions were converted to ‘I’ by RNA editing, there would be 565787 non-synonymous changes versus 137252 synonymous ones. In a striking contrast to types I and II, the frequency for non-synonymous editing events in type III, 1.82E-3 (1029/565787) is greater than that for synonymous editing, 1.47E-3 (202/137252). The increase in the frequency for non-synonymous editing is statistically significant (*Chi-squared test*, *p-value* <6.575E-3) (Fig 3C), indicating non-synonymous editing events in type III genes are advantageous and favored by positive selection through evolution.

#### Selection on type III editing events contrasting to that on gene coding sequences

While there is no established method to directly evaluate adaptation resulting from A-to-I RNA editing, we tried to gauge the effect of RNA editing by comparing selection on A-to-I editing sites with selection on genes’ entire coding sequences, and on sequences near editing sites. The selective pressure on type II and III genes was analyzed using the *Ka*/*Ks* value (the ratio of non-synonymous nucleotide substitution rate to the synonymous substitution rate) [55], which is an important measurement of functional constraints in coding gene evolution. Their *Ka*/*Ks* values (both median values smaller than 1) indicate most types II and III genes are subject to purifying selection (Fig 3D). Notably for the type III events, the positive selection on non-synonymous editing events forms contrast to the purifying selection on their coding sequences. The *Ka*/*Ks* values for the local neighbor sequences near A-to-I editing sites were calculated with sliding windows. The results (Fig 3E) indicate that the local regions near A-to-I editing sites are subjected to purifying selection (*Ka*/*Ks* <1) for either type II or III events, in accordance with those observed from the whole gene level. Genomic coding SNPs near A-to-I editing sites have similar synonymous/ non-synonymous patterns for types II and III events (S3 Fig), consistent with purifying selection in local regions around the editing sites. The presence of positively selected type III events in whole genes and local regions both under purifying selection has some special functional importance. We postulate that coding plasticity (enabled by RNA editing) creates heterozygosity in expressed genes, which confers adaptive advantage, i.e. in the cases of type III events. Note that such heterozygosity cannot be sustained by the ‘A’ and ‘G’ alleles in a diploid mating system. We reason that positive selection for RNA editing events is positive selection for heterozygosity. ‘Positive selection for heterozygosity’ enabled by A-to-I editing represents a novel selection avenue, in complement to the classic positive/purifying selection scheme. The different selective constraints between the type IIIs and others have a significant functional ramification, which is highlighted next in the contexts of tissue differentiation and development in *Drosophila*.

### Positive selection on type III editing is likely associated with nervous/synaptic activities in *Drosophila*

**Fig 4:**
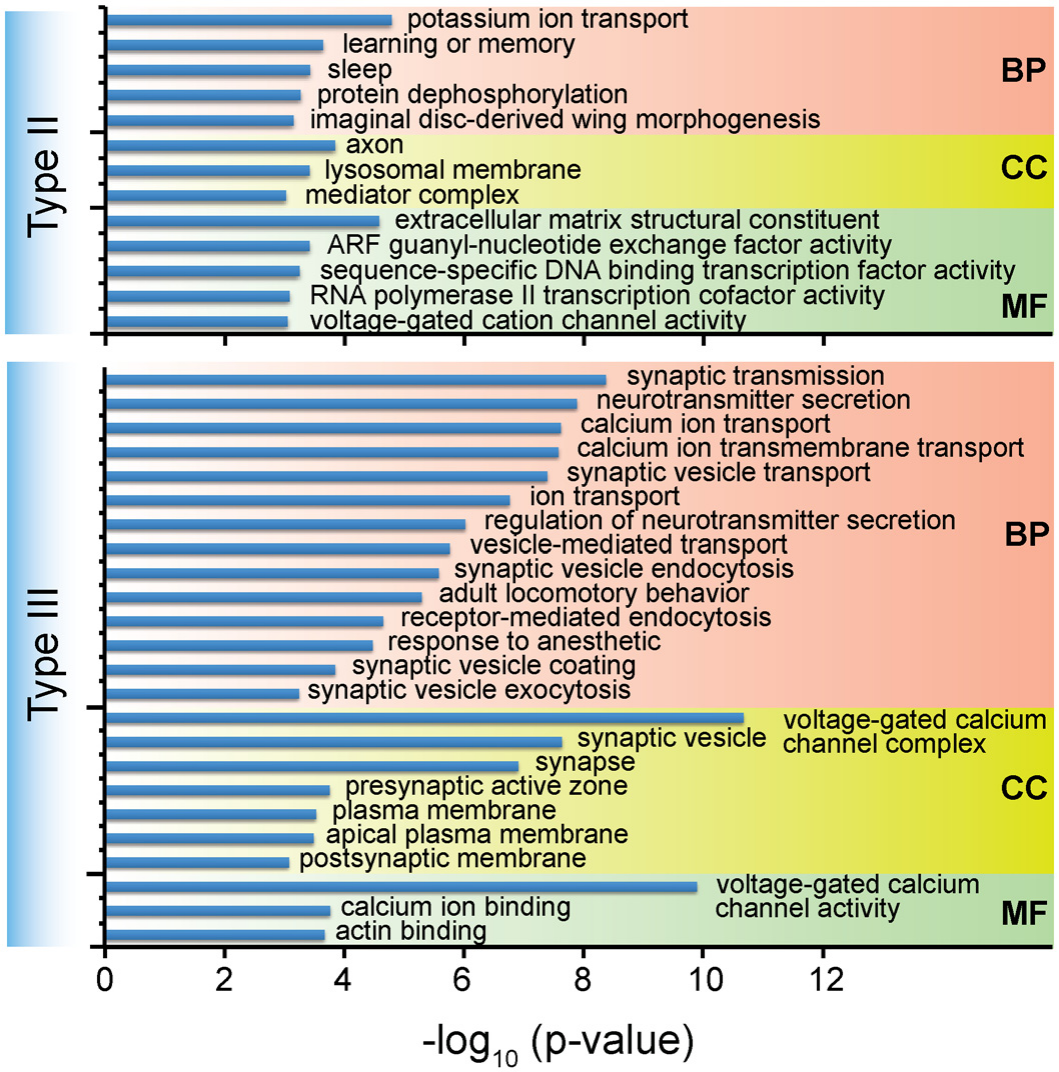
Gene ontology (GO) enrichment analysis on different types of A-to-I RNA editing events from *D*. *melanogaster*. Analyses were performed on genes of each editing type with the GOseq package [56] using the *Hypergeometric test* with *p-values* adjusted by false discovery rate (FDR) control procedure [57]. GO terms with adjusted P-values <0.001 are presented. There is no enriched GO term for type I editing events.

To understand what biological processes and functions are involved in by different A-to-I editing types, we performed Gene Ontology (GO) enrichment analysis on the genes of three editing types in *D*. *melanogaster* (see Methods for details). There was no GO term reaching the significance threshold (p-value <0.001) for the type I events. For the types II events, the top enriched GO categories were potassium ion transport (p = 1.6E-5), extracellular matrix structural constituent (p = 2.6E-5), axon (p = 1.4E-4), learning or memory (p = 2.2E-4), sleep (p = 3.7E-4), ARF guanyl-nucleotide exchange factor activity (p = 3.8E-4), and lysosomal membrane (p = 3.8E-4) (Fig 4 and S13 Table). For the type IIIs, the top GO categories were voltage-gated calcium channel complex (p = 2.2E-11), voltage-gated calcium channel activity (p = 1.3E-10), synaptic transmission (p = 2.3E-9), neurotransmitter secretion (p = 1.3E-8), synaptic vesicle (p = 2.3E-8), calcium ion transport (p = 2.3E-8), synaptic vesicle transport (p = 4.1E-8), synapse (p = 1.25E-7), and so on (Fig 4 and S13 Table). Notably, the top 13 GO categories for type IIIs had significant p-value ranging from 10^−11^ to 10^−5^, whereas the top 6 GO terms for type IIs had p-value between 10^−5^ and 10^−3^. The type III events have far more significant GO categories than type IIs, and are almost exclusively concentrated in the functions, components and processes of the nervous system. Similar analyses were also performed with other fly species (S13 Table), and the results resembled that of *D*. *melanogaster*. To further strengthen the functional relevance of A-to-I RNA editing, we further investigated the protein domains where A-to-I editing events are located. Our results indicated that type III events were significantly concentrated in functional domains (*Hypergeometric test* with *p-value* adjusted by FDR; *p-value* = 1.74E-38), whereas type I (FDR adjusted *p-value* = 1.0) and II (FDR adjusted *p-value* = 0.049) events were not significant. Looking more closely, type III event-enriched domains/families were heavily related to ion-channel function, including Ion_trans (FDR adjusted p-value = 4.39E-30), Neur_chan_LBD (FDR adjusted p-value = 9.56E-12), Neur_chan_memb (FDR adjusted p-value = 1.75E-08), and Myosin_head (FDR adjusted p-value = 8.87E-08) (S15 Table). In light of type III events being the only type subjected to positive selection, the functions of the nervous system may play a unique role in the selection and evolution of type III editing events.

### Tissue bias for different types of A-to-I RNA editing events

**Fig 5:**
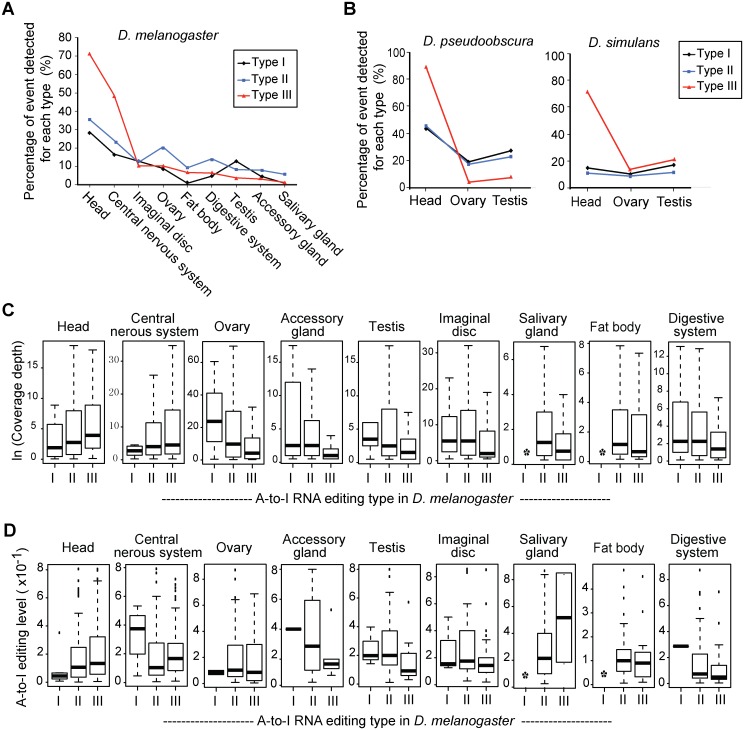
The tissue profiles for different types of A-to-I RNA editing events. (A) The percentages of A-to-I editing events detected for each type in the *D*. *melanogaster* tissues. The results were computed (see Methods for details) using tissue-specific RNA-Seq data (S2 Table). (B) The percentage of A-to-I editing events detected for each type in the *D*. *pseudoobscura* and *D*. *simulans* tissues. (C) Box plots of the expression abundance (represented by ln-transformed coverage depth) of type I, II, and III genes in the *D*. *melanogaster* tissues. *indicates no gene expression detected. (D) Box plots of the editing levels for each type in the *D*. *melanogaster* tissues. *indicates no event detected.

Given the functional bias of different A-to-I editing types and the differential selection imposed during evolution, we further looked into their tissue distribution patterns for coordinated evidence about specialization of editing types. We analyzed the transcriptome data sets from modENCODE of nine tissue types for *D*. *melanogaster*, and of three tissue types for both *D*. *pseudoobscura* and *D*. *simulans* (S12 Table; and see Methods for details). The editing events of each type were plotted in the *D*. *melanogaster* tissues (Fig 5A). For the type III events, a large majority was detected in the head and the central nervous system, and a small fraction in the other tissues. The occurrence of type I and II events was also elevated slightly in the brain tissues in *D*. *melanogaster*. Similar pattern was also supported by the tissue transcriptome data available from *D*. *pseudoobscura* and *D*. *simulans* (Fig 5B). It is likely that such pattern is held true in other fly species, whose data are limited so far. In agreement with the GO enrichment analysis, these results point to the importance of type III events in brain functions.

The gene expression abundance and the editing level for each editing type were further analyzed in *D*. *melanogaster*. The median expression abundances in the head and the central nervous system for type III genes were higher than for either type I or II events. Such trend was reversed in all the other tissue types (Fig 5C). The median editing levels in the head and the central nervous system were also higher for type III events than for either type I or II events, with the exception of the central nervous system, where a small number (only 4) of type I events were counted (Fig 5D). However, the median editing levels for type III events were mostly lower in the rest tissue types.

Taken together, the type III genes were preferentially expressed and edited in the head and central nerve system. Although biased distribution of A-to-I editing events toward brain tissues was previously reported in rhesus [22], mice [20], and human [43], we showed for the first time that preference was established toward a fraction of the editing events (type III), which were subjected to positive selection associated with nervous/synaptic activities in *Drosophila*. It is likely that other event types occurring in brain tissues are the by-products of A-to-I RNA editing machinery. On the other hand, although positive selective constraint on type III editing events is overwhelmingly concentrated in the components and functions of the nervous system, we cannot rule out that other functions and processes drive adaptive selection on A-to-I editing events. The high expression abundance and high editing level for some events in the non-brain tissues hint on such possibility (Fig 5C and 5D).

### Changing patterns of different editing types during holometabolous development and in mating response in *Drosophila*

**Fig 6:**
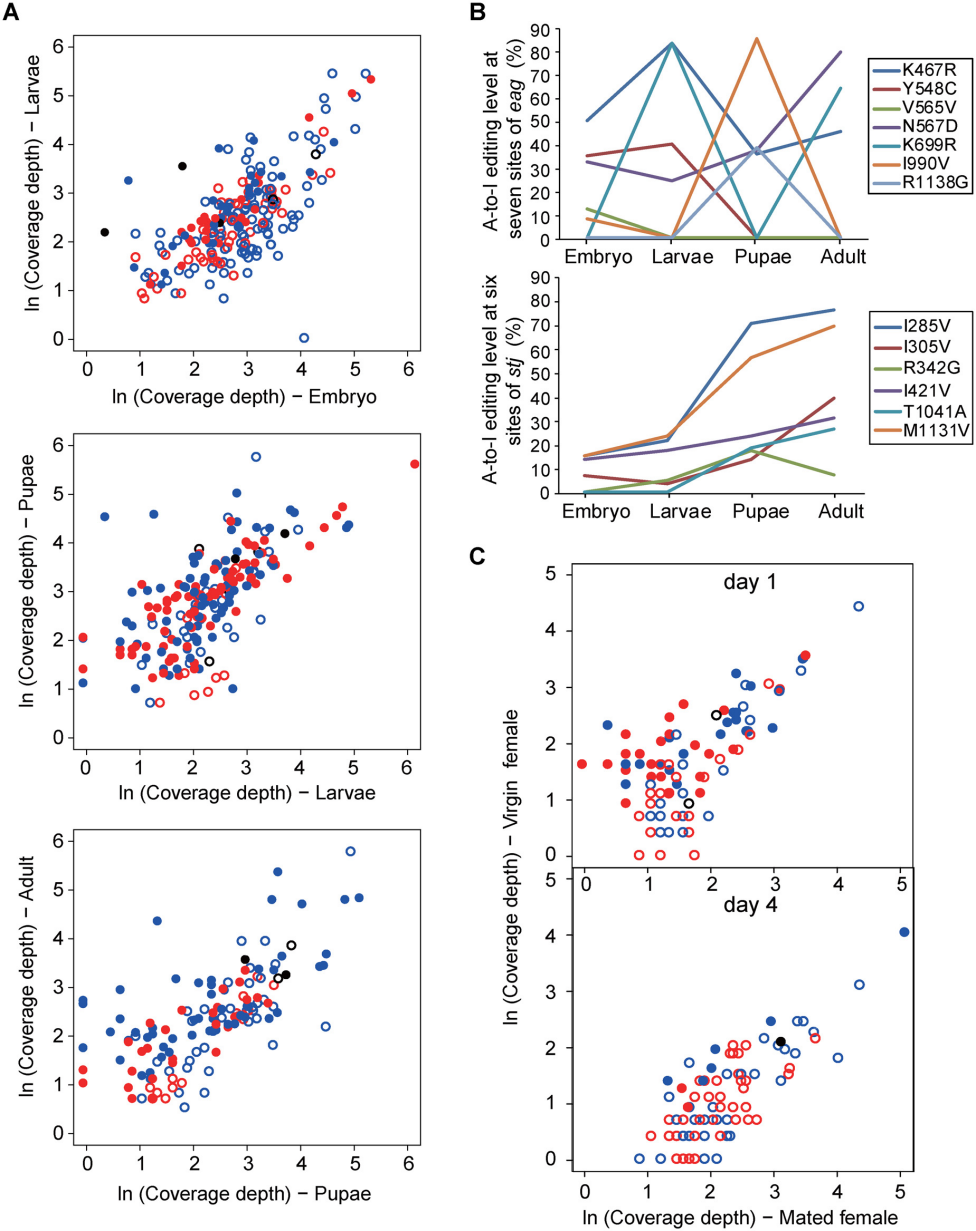
Changes in different types of A-to-I editing events in holometabolous development and post-mating response in *D*. *melanogaster*. (A) Changes in different types of editing events between embryo and larvae (top), between larvae and pupae (middle), and between pupae and adult (bottom). The events were detected as described in *Methods*, using development-specific RNA-Seq data (S3 Table). The expression abundance of edited genes is presented as ln-transformed coverage depth. Open circles represent editing events detected only in the stage corresponding to the X-axis; filled circles only in the stage corresponding to the Y-axis. The event type is denoted by the color: black for type I, blue for type II, and red for type III. (B) Shifting of editing levels for *eag* and *stj* transcripts at multiple sites during holometabolous development. (C) Changes in different types of editing events in post mating response. The editing events different between virgin and mated females are illustrated for days 1 and 4 post-mating. The events were detected as described in *Methods*, using head RNA-Seq data from virgin and mated female flies (S2 Table). Open circles represent editing events detected only in virgin females (X-axis), and filled circles only in mated females (Y-axis). The event type is denoted by the color scheme as same as in 6A.

To understand the physiological significance of different editing types, we investigated their patterns in two important aspects of fly life cycle: holometabolous development and mating response. First, the occurrences of A-to-I editing events at the four developmental stages in *D*. *melanogaster* were analyzed. Embryo, larvae, pupae, and adult shared 133 common editing events (in 96 genes), with 37 and 93 being type II and III, respectively (S10 Table). Considerable changes in A-to-I editing happened between embryo and larvae, between larvae and pupae, and between pupae and adult (Fig 6A). For example, 2, 105, and 50 disappeared, and 3, 27, and 24 emerged for type I, II, and III events, respectively, in transition from embryo to larvae. They included a type II event on Npc1a (Niemann-Pick C1 protein) gene that was lost, and a type III event on *Rdl* (glycine receptor alpha-3) that emerged. Also note shift in gene expression levels accompanied some of the changes in editing events (Fig 6A).

The shifting patterns of different editing types during holometabolous development illustrated the dynamic and active nature of A-to-I RNA editing, which are exemplified by *eag* and *stj* genes in *Drosophila*. *eag* encodes a voltage-gated potassium channel, for which A-to-I editing could alter amino acid in the critical S6 segment and the cytoplasmic C-terminal domain for binding cyclic nucleotide. We observed a striking pattern in changes of RNA editing level on seven sites throughout fly life cycles (Fig 6B, top panel). Similar patterns on four of these sites were previously reported [58]. The RNA editing-induced changes on *eag* potassium channel were found to modulate its activation kinetics in *D*. *melanogaster* [58]. In contrast, the *stj* (*straightjacket*) gene, which encodes the alpha(2) delta subunit of the voltage-gated calcium channel in neurons, exhibited a different editing pattern (Fig 6B, bottom panel). As a critical component involved in the neuromuscular junction development, synaptic transmission, and synaptic vesicle endocytosis [59–61], this represents the first reported finding on the editing pattern of *stj* transcripts. We postulate that *eag* and *stj* proteins acquire a host of fine-tuned channel property through A-to-I editing with the combination of multiple sites at variable editing levels. The resulting diversity of *eag* and *stj* proteins enables a wide range of excitability and complex regulation in fly nervous system.

Second, to investigate whether and to what extent the different types of A-to-I editing events are involved in post-mating response in flies, we analyzed the published RNA-Seq data from paired virgin and mated female flies (S2 Table). Mating is known to induce profound physiological and behavioral changes in the female flies. The so-called long-term post-mating changes usually last about a week, involving changes in the expression of hundreds of genes in brain tissues [62,63]. Comparing the A-to-I editing events in the head tissues, significant changes in different editing types were observed between day 1 virgin and mated females, and between day 4 virgin and mated females (Fig 6C and S11 Table). Notably, the changes in RNA editing in mated females concentrated in synaptic receptors and ion channels, e.g. synaptotagmin-1, endophilin-A, glycine receptor alpha-3, ryanodine receptor-2, voltage-dependent calcium channel (beta), etc. To our knowledge this is the first reported observation that implies that A-to-I RNA editing is actively involved in the post-mating response in *Drosophila*.

## Discussion

A-to-I RNA editing adds a critical layer of functional modulation on genes and has been recognized as an important mechanism to expand the genetic repertoire through coding plasticity. The extent of impact of A-to-I editing on the diversity of transcriptome and proteome, and the selective constraint imposed on RNA editing events through evolution are some of today’s key issues in evolutionary biology. Our study was designed to take advantage of the large collection of genome and transcriptome sequencing data that were only available recently. The analysis was performed using the combination of two dimensional data sources: fly species across a defined evolutionary timeframe, and tissue samples across a range of tissue types and developmental stages. The evolution of the A-to-I editing events in *Drosophila* was revealed with some important observations. First, A-to-I RNA editing on coding genes is confined to a relatively small group of transcripts in the *Drosophila* phylogeny. Conservatively, about 5% of coding gene families in *Drosophila* are targeted by A-to-I editing. The majorities of A-to-I editing events are not conserved between homologous genes. Second, based on the conservation of A-to-I RNA editing sites, there appears to be three distinct types of editing events on genes’ coding regions, corresponding to the editing events of different ages. While the type I and IIs are presumably young non-conserved editing events in singleton genes or in multi-member gene families, respectively, type IIIs are conserved events in multi-member gene families. For the majority of editing events, i.e. type IIs, non-synonymous substitutions are deleterious and purged by purifying selection. In contrast, the type III events are driven by positive selection, where non-synonymous changes are preserved. Third, the type III events were found to be concentrated in the head tissues, and highly enriched in a narrow range of components and functions of the nervous system (Figs 4 and 5). The results from enrichment analysis of type IIIs and its biased distribution suggest that the positive selection on type IIIs is associated with their involvement in the nervous/synaptic activities. While many A-to-I editing cases were reported by others to occur in the nervous system [36,37,64], there has not been evidence like ours to show that a clear portion of editing events (type III) being positively selected during evolution, are overwhelmingly associated with the nervous system/brain functions. And equally importantly, a larger portion of editing events (type I and II) being under purifying selection, do not have such association. Forth, the patterns of different event types were found to shift between developmental stages and in post-mating response in female flies. The data suggest that the A-to-I RNA editing is actively involved in these processes, underlain by a complex regulation of A-to-I RNA editing in flies. The rapid shifts in A-to-I editing can modulate gene function dynamically, having a profound implication for fast acclimatization and rapid response to changing environmental conditions.

The adaptive potentials of A-to-I RNA editing are the subject of intense debate over the past years. On one hand, un-controlled editing events can disturb or disrupt the normal gene function networks, hence reducing the fitness of living organisms. On the other hand, RNA editing offers genes coding plasticity that can be advantageous in evolution. The competing probabilities are summarized by the ‘continuous probing’ model [28]. Under this model, new low-level editing events emerge at many sites continuously, which forms the molecular basis for adaptability through continuous selection. Such pool of varying editing sites may confer acclimatizing and adaptive advantage for organisms in changing environments, representing an enhanced evolvability with a low cost in fitness as the un-edited bases are also present to function under normal conditions [28]. Our analysis of A-to-I RNA editing events in flies adds new details to the subsequent process of natural selection. It appears that the non-synonymous A-to-I editing, in general, is rather deleterious. The majority of editing events, i.e. type I and IIs, are driven by purifying selection, in which non-synonymous events are purged (Fig 3B and 3C). The selection mechanism mostly likely operates at the organism level where individuals with detrimental non-synonymous editing events are counter-selected. It is also possible that such counter-selection happens within the cell at the molecular level, but it is a less likely mechanism, as no clear case has been found in support of it. In addition, the neutral non-synonymous editing events, if ever exist, would account for a very small fraction. A-to-I RNA editing observed in our study appeared in general to impose some burden on fitness.

On the other hand, a minority of editing events, i.e. type IIIs, are driven by positive selection, which are conserved in homologous genes and preserved across multiple species. These beneficial events are concentrated mainly in functions and components of the nervous system. Although a few cases of beneficial A-to-I editing outside of neuronal receptors and brain-specific ion channels were documented by different researchers [7,24,65–67], there was little indication that editing events outside of the nervous system are adaptive, which is contrasting and surprising (Fig 4). It appears that nervous system functions are mainly tested to determine if an A-to-I editing is beneficial for an organism. Underlying our conclusion, it was suggested that in the brain the broadened diversity of the transcriptome created through A-to-I RNA editing may be part of the process in memory-formation [28]. Coincidentally or not, the oldest ADAR enzymes arising at the beginning of metazoan lineage, accompanied the occurrence of the most primitive nervous system in animals [68]. Our analysis provided a thorough account about the type III events being highly involved in the nervous functions and processes. Previously, the consequences of RNA editing deficiency were revealed by the *ADAR* mutant flies, which displayed a phenotype of severe behavior dysfunction and neurological defects in the central nervous system [17]. The severe alterations in synaptic ultrastructure and the impaired synaptic release at larval neuromuscular junctions was identified as the cause for defects in synaptic development and for dysfunctions from motility to courtship in *ADAR* mutant flies [69]. In addition, our work found changes of different editing types occurred throughout the developmental cycles and in post-mating response in *Drosophila* (Fig 6), implying the active involvement of A-to-I editing in development and in physiological activities. Supporting our finding at the transcriptome level, individual editing sites were found by previous studies to be developmentally regulated in flies[3,70] and in mammals [71,72].

Why is the beneficial effect of A-to-I editing observed with the type III events largely limited to the central nervous system in flies, but not in a broader spectrum of biological processes or functions? While answer to this intriguing but difficult question remains elusive to us, we may speculate that the coding plasticity enabled by A-to-I RNA editing generates a new class of binary variations that uniquely fit the property required for functioning by the animals’ central nervous system. It is possible that ion channels of heterogeneous composition created by RNA editing have become intrinsic components of the functional nervous system. It is also apparent that the ability to fine-tune ion channels and receptors by A-to-I editing cannot be supported by the ‘A/G’ heterozygote, as it is almost impossible to sustain such heterozygosity in all offspring through the diploid mating system. So the A-to-I RNA editing scheme is an effective alternative to maintain heterogeneous components of the nervous system. While we could not rule out the cases of adaptive A-to-I editing that are driven by positive selection from activities outside the nervous system, their restriction mostly to the nervous system is somewhat puzzling. One possible explanation could be that outside the nervous system the benefit of amino acid substitutions from A-to-I recoding is limited, which cannot offset their deleterious effect through evolution.

In summary, with the extensive data collections from seven fly species spanning a defined phylogenetic distance, we systematically characterized their A-to-I RNA editome, establishing the prevalence of A-to-I editing and the extent of impact on transcriptome. We further unraveled the evolutionary dynamics of RNA editing events by deriving their time-course of events from closely related species. Importantly, we have shown that A-to-I editing events in CDS regions are grouped into three distinct types based on the conservation of the editing sites. Although A-to-I editing events in general are deleterious, a minority of events (type III) that are subjected to positive selection, are mostly associated with the components and function of the nervous system. Tissue specific profiles of the RNA editing types and their changes during holometabolous development and in post-mating response reveal the dynamic nature of A-to-I editing, which points to an underlying mechanism for complex regulation. In essence, the potential of genetic diversity and complexity created by A-to-I RNA editing, and their impact on various bio-physiological processes are shaped and realized by the balance between positive selection on beneficial editing events and the purifying of detrimental ones.

## Materials and Methods

### Collection of genome and transcriptome sequencing data

The modENCODE projects are the main source for the *Drosophila* data used in this study. It is complemented by additional data from NCBI Sequence Read Archive (SRA; http://www.ncbi.nlm.nih.gov/sra) and from NCBI Gene Expression Omnibus (GEO; http://www.ncbi.nlm.nih.gov/geo/). More details on the sequencing data are found in the S1–S3, S6 and S12 Tables.

The whole-fly transcriptome sequencing data for the *Drosophila* species, *D*. *ananassae*, *D*. *melanogaster*, *D*. *mojavensis*, *D*. *pseudoobscura*, *D*. *simulans*, *D*. *virilis*, *D*. *yakuba*, were obtained from modENCODE project: Transcriptional Profiling of additional *Drosophila* species with RNA-Seq (Lab: Brian Oliver) (S1 Table). The tissue transcriptome sequencing data for *D*. *melanogaster* were obtained from modENCODE project: Tissue-specific Poly(A) Site Profiling of *D*. *melanogaster* using Illumina poly(A)+ RNA-Seq (Lab: Brenton Graveley) (S2 Table). The developmental-stage transcriptome sequencing data for *D*. *melanogaster* were obtained from modENCODE project: Developmental Time Course Transcriptional Profiling of *D*. *melanogaster* Using Illumina poly(A)+ RNA-Seq (Lab: Brenton Graveley) (S3 Table). The transcriptome sequencing data for *D*. *melanogaster* pharate adult dataset [40] used for validation was obtained from NCBI GEO under accession number GSE50711. The head transcriptome sequencing data for the *Adar*^*5G1*^ mutant and paired wild type *D*. *melanogaster* strains *w*^*1118*^ were obtained from NCBI SRA under accession numbers: SRR629969 and SRR629970 [36]. The tissue transcriptome sequencing data for *D*. *pseudoobscura and D*. *simulans* were obtained from modENCODE project: Transcriptional Profiling of additional *Drosophila* species with RNA-Seq (Lab: Brian Oliver) (S12 Table), and from NCBI GEO under accession numbers: GSM1258036, GSM1258037, GSM1258038, GSM1258039, GSM1258040, GSM775506, GSM775507, GSM775508, GSM775509, GSM775510, GSM1306668, GSM1306669, GSM1306670, and GSM1306671. The genome re-sequencing data for *D*. *melanogaster* were obtained from NCBI SRA under accession numbers: SRR485845, SRR485846, SRR485847 [10], SRR1516226 (BioProject PRJNA244953), and from modENCODE project: Genome assembly and alignment of *D*. *melanogaster* OreR virgin female from Bloomington stock to reference r5 (Lab: Brenton Graveley; DDC id:modENCODE_5518).

For analysis, the reference genomes and gene annotation data for *Drosophila* species, *D*. *ananassae* (r1.3), *D*. *melanogaster* (r5.53), *D*. *mojavensis* (r1.3), *D*. *pseudoobscura* (r2.29), *D*. *simulans* (r1.4), *D*. *virilis* (r1.2), *D*. *yakuba* (r1.3) were downloaded from the FlyBase (ftp://ftp.flybase.net/genomes/). Those for *A*. *aegypti* (AaegL1.3, April 2012) were obtained from Vectorbase (https://www.vectorbase.org).

### Sequence mapping and pipeline for identification of A-to-I RNA editing

The raw sequencing data were first processed to remove low quality reads. The sequencing reads were trimmed from both the 5’ and 3’ ends, with a quality score threshold of 20, using program Sickle (version 1.33) [73]. Any reads containing N were also removed. The consequential clean datasets were evaluated with FastQC (http://www.bioinformatics.babraham.ac.uk/projects/fastqc/). The pipeline for identification of A-to-I RNA editing was modified from what was used in Ramaswami’s work [36]. First, quality RNA-Seq reads from each species were mapped to their genomes using Burrows-Wheeler algorithm [74], employed by Tophat program (version 2.0.8b) [75] with the parameters ‘-G reference.gtf’ and ‘-N/—read-mismatches’ set to 3. The reference genomes and related gene models for the *Drosophila* species were retrieved from FlyBase as described in section: *Collection of genome and transcriptome sequencing data*. Second, the RNA variances were called using Samtools (Version: 0.1.13) [76] pileup program with options”-Q 15”. The resulting variant bases were reported with the numbers of reads supporting either the reference genotype or the variance genotypes. Third, the RNA variances were filtered using the following criteria to identify A-to-I editing events: 1) variant sites with coverage depth > = 5; 2) variant sites located over 10 bp away from either end of a sequence read; 3) variant sites with > = 2 non-identical supporting reads; 4) variance rate between 1% and 90%; 5) occurring in at least 50% of all samples for a species; 6) retaining only A-to-G base changing events.

### Estimating genuine A-to-I RNA editing events by comparing head data from wild type and *ADAR*-mutant flies

A-to-I RNA editing is catalyzed by the enzyme ADAR, and A-to-I editing events were found to be abolished in *ADAR*-mutant flies. To validate the identified A-to-I editing events and estimate the rate of false positives, we sampled the events occurring in the heads of day 5 wild type (*w*^*1118*^) fly, and compared with those from the heads of day 5 *Adar*^*5G1*^ mutant [36]. The transcriptome sequencing data from day 5 wildtype fly and day 5 *Adar*^*5G1*^ mutant fly were processed, mapped and filtered as described in the section: *pipeline for identification of A-to-I RNA editing*. For those A-to-I editing events found to occur in the heads of day 5 wild type flies, their corresponding nucleotide resides in the heads of day 5 *Adar*^*5G1*^ mutant flies were examined. Those that were found to be adenosine residues only in *Adar*^*5G1*^ mutant flies are considered genuine A-to-I RNA editing events.

### Estimating the false positive rates of A-to-I editing events due to genomic variants

We first created a *D*. *melanogaster* genomic variant database (S1 Text) by combining SNP data from FLYSNPdb [34] with the genomic variant data we identified from the *D*. *melanogaster* genome re-sequencing data. Excluding INDELs and other types of polymorphisms, the FLYSNPdb comprised more than 21307 SNP that were imported into our database. In addition, we isolated SNPs using three sets of genome re-sequencing data (described in the section: *Collection of genome and transcriptome sequencing data*) with our SNP pipeline. Briefly, the sequencing reads were mapped to the *D*. *melanogaster* genome (r5.53) using bowtie2 (version 2.1.0) [74] with options “-N 1”. The base variances were called using Samtools (Version: 0.1.19) [76] mpileup program with options”-Q 20”. The resulting base variants were further filtered with following parameters: 1) variant sites with coverage depth > = 5; 2) variant sites located over 10bp away from either end of a sequence read; 3) variant sites with > = 2 non-identical supporting reads; 4) variance rate >1%.

To identify genomic variants that match an A-to-I RNA editing event, we first filtered the *D*. *melanogaster* genomic variant database and retained only A-to-G base changing sites. The resulting A-to-G SNPs were compared with *D*. *melanogaster* A-to-I RNA editing sites. Any A-to-I editing site matching a genomic A-to-G SNP was suspected to be resulted from a genomic variant.

### Validation of A-to-I RNA editing events with Sequenom’s MassARRAY platform

For experimental validation, the samples of six fly species, *D*. *ananassae*, *D*. *mojavensis*, *D*. *pseudoobscura*, *D*. *simulans*, *D*. *virilis*, and *D*. *yakuba*, were ordered from the *Drosophila* Species Stock Center at the University of California, San Diego, whereas the samples of *D*. *melanogaster* were obtained from Core Facility of *Drosophila* Resource and Technique, Institute of Biochemistry and Cell Biology, Chinese Academy of Sciences, Shanghai. For each species 20–30 fly individuals were pooled before gDNA and total RNA were extracted in parallel. The gDNA was isolated according to the protocol of VDRC stock center (http://stockcenter.vdrc.at/control/protocols). The total RNA was extracted using RNeasy kit (Qiagen, Germantown, MD, USA) and cDNA was synthesized using RevertAid First Strand cDNA Synthesis Kit (Thermo Scientific, Waltham, MA, USA), according to the manufacturers’ instructions. Thirty to thirty-five A-to-I editing sites were randomly chose for each species, with twenty to twenty-five from CDS regions and ten from non-CDS regions. Genotyping was performed on reverse-transcripted cDNA and matching gDNA using the iPLEX Gold Assay (Sequenom, San Diego, CA, USA). Assay primers were designed with the MassARRAY Assay Design software (version 3.1; Sequenom). Allele specific extension was performed with iPLEX Gold reagent kit (Sequenom). Extension products were subjected to MALDI-TOF mass spectrometry (MassARRAY Analyzer Compact; Sequenom), according to the manufacturer’s instructions. Genotypes were automatically called using the MassARRAY Typer software (Sequenom), and checked manually. Genotyping results from cDNA and matching gDNA were compared and positive events were confirmed with ‘G’ allele found in cDNA (G/Total > = 0.10) and ‘A’ allele found in gDNA (A/Total >0.90), as described by Chen et al [22].

### Annotation of A-to-I RNA editing sites in *Drosophila*

A-to-I RNA editing sites were annotated with ANNOVAR [77] using gene models from FlyBase for the *Drosophila* species, *D*. *ananassae*, *D*. *melanogaster*, *D*. *mojavensis*, *D*. *pseudoobscura*, *D*. *simulans*, *D*. *virilis*, and *D*. *yakuba*. A-to-I RNA editing sites were annotated with gene definitions, including CDS, intronic, 5’UTR, 3’UTR, and intergenic. Those within coding regions (CDS) were further defined as “synonymous” or “non-synonymous” based on whether they change the amino acid in protein products.

Because the gene models for *D*. *yakuba*, *D*. *ananassae*, *D*. *simulans*, *D*. *mojavensis* and *D*. *virili* lack the untranslated regions (UTR) structure definition for genes, we had to first define their UTR structures as described in the section: *Refining UTR regions in Drosophila*. We then combined the refined UTR structures with the FlyBase gene models of the five species, which was used in annotation by ANNOVAR.

### Refining UTR regions in five *Drosophila* species

The UTR structures for *Drosophila* species, *D*. *yakuba*, *D*. *ananassae*, *D*. *simulans*, *D*. *mojavensis* and *D*. *virili*, were defined with the help of available trancriptome sequencing data. The sequencing reads from whole-fly transcriptome data (S1 Table) were first mapped to the reference genomes of *D*. *ananassae* (r1.3), *D*. *mojavensis* (r1.3), *D*. *pseudoobscura* (r2.29), *D*. *virilis* (r1.2), and *D*. *yakuba* (r1.3) with Tophat (version 2.0.8b). The coverage depth for mapping sequences was reported with Samtools (Version: 0.1.13) and BEDTools (Version: 2.12.0). Then their corresponding gene models (CDS) acquired from FlyBase were superimposed to their genome, before the CDS regions were extended upstream and downstream based on mapped reads. The maximum lengths for 5’UTR and 3’UTR were set at 600 bp and 1400 bp, respectively. The parameters were chosen because 95% of 5’UTRs were within 600 bp upstream of translation initiation codons, and 95% of 3’UTRs were within 1400 bp downstream of stop codons in the *D*. *melanogaster* gene models. The refined UTRs for gene models in the five species, *D*. *yakuba*, *D*. *ananassae*, *D*. *pseudoobscura*, *D*. *mojavensis* and *D*. *virili*, are available in S2 Text.

### Profiling gene expression abundance and A-to-I RNA editing levels

The transcriptome sequencing data were processed and mapped to reference genomes as described in the section “*Sequence mapping and pipeline for identification of A-to-I RNA editing*”. The mapping files were processed with Cufflinks (v2.1.1) [45] with options “-g *.gff” to estimate the gene expression for nine *D*. *melanogaster* tissue types. FPKM (fragments per kilobase of transcript per million mapped reads) was used to measure the gene expression abundance. The editing levels for A-to-I editing sites were estimated using the Samtools (Version: 0.1.13) pileup program, which reported the numbers of reads supporting either the reference genotype or the edited genotype. The editing level for each site was calculated as percentage of reads in edited genotype out of total reads mapped to the site.

### Detection of A-to-I RNA editing events and hierarchical clustering analysis across tissues

The tissue and development stage transcriptome sequencing data (S2 and S3 Tables), including the brain tissue RNA-Seq data from virgin and mated female individuals, were processed and mapped to reference genomes as described in the section “*Sequence mapping and pipeline for identification of A-to-I RNA editing*”. The *D*. *melanogaster* RNA editing sites from the reference list were scanned, and the numbers of reads supporting either the reference genotype or the edited genotype were reported and analyzed. Only the events meeting the following criteria were designed to be present in a tissue sample: 1) variant sites having coverage depth > = 5; 2) variant sites having at least 10 bp from either end of a sequence read; 3) variant sites with at least two non-identical reads supporting edited genotype.

The hierarchical clustering was performed by first building a matrix based on the presence/absence of A-to-I editing events in the nine different tissue types for all *D*. *melanogaster* editing sites from the reference list. The matrix was processed with heatmap function from R (http://www.r-project.org/) using “complete hierarchical cluster” algorithm and option “distfun = dist(method = ‘euclidean’)”.

### Computing secondary structure minimum free energy for RNA editing sites

To calculate the secondary structure minimum free energy for A-to-I RNA editing sites, we first extracted 60 bp sequences flanking the editing sites (30 bp upstream and 30 bp downstream). The secondary structures for the 61 bp sequences for all sites were built using RNAFold (2.0.7) from ViennaRNA Package 2.0 [50] with options “—temp = DOUBLE;—dangles = 2;—noGU”, and the minimum free energy for the folding structures was calculated. As a control, random 61 bp CDS regions from 2000 arbitrarily picked *Drosophila* genes were isolated, and their secondary structures were predicted using the same protocol with minimum free energy computed as described above.

### Phylogenetic analyses of A-to-I editing events and host genes

To study the conservation of coding genes targeted by A-to-I RNA editing in *Drosophila*, the homologous gene families were constructed. The entire gene sets from the seven species, *D*. *ananassae* (r1.3), *D*. *melanogaster* (r5.53), *D*. *mojavensis* (r1.3), *D*. *pseudoobscura* (r2.29), *D*. *simulans* (r1.4), *D*. *virilis* (r1.2), *D*. *yakuba* (r1.3) were downloaded from the FlyBase. The OrthoMCL pipeline [78] was used to cluster encoded gene products into homologous families, as previously described [79]. Briefly, poor quality coding sequences were filtered using the orthomclFilterFasta module with options “min_length = 10; max_percent_stop = 20”. Then BLAST search with blastp was conducted with the option “–e 1E-5” (E value threshold). Clustering with MCL module was performed with options “-abc” and “-i 5.0”. The proteins from the seven *Drosophila* species formed 30,434 families, and among them 10,820 contained more than one member.

Using the clustered homologous gene families from the *Drosophila* species as reference, the identified A-to-I edited genes were mapped into families. A total of 1,526 gene families comprised genes with A-to-I editing events; of which, 133 were singleton genes (8.72%) and 1393 were multi-member gene family (91.28%). Based on the conservation of RNA editing sites, the CDS events were categorized into three types. The type I events occurred in singleton genes that did not have detectable homologous gene in other fly species. The type II events were non-conserved editing events in multi-member gene families, but each occurred in one member and had no conserved event in other members of the same family. The type III events referred to conserved editing events occurred in at least two members of a multi-member gene family.

We investigated the event gains and losses of type III events along the phylogeny using the Gain Loss Mapping Engine (GLOOME) [52] (http://gloome.tau.ac.il/). (Since each of type I or II events is only present in one terminal leaf of the phylogenetic tree, it is not necessary to include them in the analysis). The type III events were grouped into 402 clusters based on conservation of editing sites. Then the presence and absence profile (phyletic pattern) was generated [52] based on the clustering of type III events. With uploaded phyletic pattern matrix of type III events, GLOOME server inferred branch specific gain and loss events along the phylogeny using stochastic mapping [53].

### Analysis of *Ka*/*Ks* on the coding genes with A-to-I editing events

The selective pressure on the coding genes targeted by A-to-I editing was analyzed using the *Ka*/*Ks* value (the ratio of non-synonymous nucleotide substitution rate to the synonymous substitution rate) [55]. The orthologous genes from *A*. *aegypti* were used as outgroup in computing *Ka* and *Ks* values. The genes harboring A-to-I editing events were paired with its orthologs from *A*. *aegypti*, which were identified using bidirectional best hits (BBH) algorithm [80]. The *Ka* and *Ks* values for each pair were computed with codeml program from PAML package, using maximum-likelihood method [81]. The *Ka* and *Ks* values were then corrected with Colbourne’s protocol [82].

To investigate the details of purifying selection on genes with type III events, the Ka/Ks values for the local neighbor sequences near A-to-I editing sites were calculated using shifting windows with a size of 11 codons. For each shifting window, the *Ka*/*Ks* value of a local sequence was computed with codeml program using the 11-codon aligned block between the local sequence and orthologous one from *A*. *aegypti*.

### Enrichment analyses of gene ontology and protein domain for different types of RNA editing events

The genes with different types of RNA editing events in *D*. *melanogaster* were compiled, and the lists of type I, II, and III genes were created, respectively (S13 Table). Gene ontology (GO) enrichment analyses were performed on genes of each editing type with GOseq package [56] from R using the *Hypergeometric test* with *p-values* adjusted by false discovery rate (FDR) control procedure [57]. A significant GO term required at least two enrichment genes and five background genes. The GO terms at the top of the tree hierarchy, namely cellular component (CC), biological process (CC), and molecular function (MF), were excluded from the significant list.

Protein domain enrichment analyses were performed on the protein domains where A-to-I editing events fall in. Genes with A-to-I editing events were annotated with domain information using Pfam webserver(v29.0) [83] (http://pfam.xfam.org/) with default parameters. The proportion of number of editing events within domains over total event number was tested against the proportion of all domain size over all gene size. Domain enrichment analyses were performed using the *Hypergeometric test* similar to that described in the GO enrichment analyses.

## Supporting Information

S1 Table The sources of deep-sequencing transcriptome data from the seven *Drosophila* species. (XLSX)

S2 Table RNA-seq data of nine different tissues of *D*. *melanogaster*. (XLSX)

S3 Table RNA-seq data of four development stages of *D*. *melanogaster*. (XLSX)

S4 Table The reference set of A-to-I RAN editing events from seven *Drosophila* species. (XLSX)

S5 Table Overlapping of our *D*. *melanogaster* subset of A-to-I RNA editing events with other studies. (XLSX)

S6 Table RNA-seq data of pharate adults of *D*. *melanogaster*. (XLSX)

S7 Table A-to-I RNA editing sites having adenosine residues only in the head of Adar5G1 mutant flies. (XLSX)

S8 Table A-to-I RNA editing sites within *D*. *melanogaster* non-coding RNA sequences. (XLSX)

S9 Table Gene families that harbor identified A-to-I RNA editing sites. (XLSX)

S10 Table Occurrences of A-to-I RNA editing events in different development stages of *D*. *melanogaster*. (XLSX)

S11 Table Shifting of RNA editing events in the head tissues between virgin and mated females of *D*. *melanogaster*. (XLSX)

S12 Table Data source information for RNA-seq from tissues of *D*. *pseudoobscura*, *D*. *simulans* and *D*.*mojavensis*. (XLSX)

S13 Table GO enrichment results for edited genes of *D*. *melanogaster* and other *Drosophila* species. The column 'numDEInCat' represents the number of editing host genes with corresponding GO terms annotation. The column ‘numInCat’ represents the number of total genes with corresponding GO terms annotation. GO enrichment tests were performed for each GO category with alternative hypothesis ‘the proportion of edited genes among all genes in one GO category is higher than the random expectation (over_represented_pvalue)’, and alternative hypothesis ‘the proportion of edited genes among all genes in one GO category is less than the random expectation (under_represented_pvalue)’, separately. We only tested these GO categories with >4 edited gene numbers.
(XLSX)

S14 Table The replication of identified editing events in two *Drosophila* species. (XLSX)

S15 Table The protein domain function enrichment analysis of editing events. Function domain/family enrichment tests for editing events were performed for each function entry annotated with Pfam (Supplemental *ref*. *1*). We tested the proportion of the number of edited domains for each Pfam entry (represented by a HMM model) against the proportion of all edited domains in all Pfam entries. We used the hypergeometric distribution to calculated the p values and then adjusted the p values with FDR method. We only tested these categories with more than 4 edited numbers.
(XLSX)

S16 Table The validation list of editing events from 7 *Drosophila* species using Sequenom MassARRAY platform. Approximately 19% of the assays yielded no signal (missed) or a signal that was inconsistent with the genomic DNA (gDNA) controls (control_failed). We excluded them from further evaluation. An A-to-I editing site was confirmed the ratio of edited form (G signal / total signals) was > = 0.10 on in cDNA samples, and <0.10 in the DNA samples, or when the odds ratio (OR) is over 3 (OR = (cDNA G signal / cDNA A signal)/(DNA G signal / DNA A signal)) that indicated a large increase of the proportion of edited forms in cDNA samples likely due to editing events. Otherwise, the events were not confirmed.
(XLSX)

S17 Table Statistic testing of A-to-I editing events being significantly biased toward genes’ CDS regions. (XLSX)

S1 Text *D*. *melanogaster* genome variance database. We created the *D*. *melanogaster* genomic variant database by combining SNP data from FLYSNPdb (Supplemental *ref*. *2)* with the genomic variant data we identified from the *D*. *melanogaster* genome re-sequencing data, including SRR485845, SRR485846, SRR485847, SRR1516226, and modENCODE_5518.
(ZIP)

S2 Text Refined UTRs for five *Drosophila* species including *D*. *ananassae*, *D*. *mojavensis*, *D*. *simulans*, *D*. *virili*s and *D*. *yakuba*. The refined UTR regions for each species were presented in gff3 format.
(ZIP)

S3 Text Supplemental references. (DOCX)

S1 Fig The proportion of all 12 base change types from 7 *Drosophila* species. The dash line above each bar represents the standard deviation of corresponding value. The blue line marks the value for *D*. *melanogaster*. We applied the same screening method for all base change types (*see* Methods), and adjusted the parameters according to the basespecific error rates from Illumina sequencing platform (Supplemental *ref*. *3*). Assuming that all non-canonical mismatches were background noise, and the error rates for all 12 base change were equal, the false positive rate for A-to-G change type was estimated to be 5.59% [(38.1%/11)/61.9% = 5.59%] for all sites, and 6.32% [(41.0%/11)/59% = 5.59%] for CDS sites (Supplemental *ref*. *4*).
(PDF)

S2 Fig Events gained and lost in *Drosophila* lineage. We used the Gain Loss Mapping Engine (GLOOME) server (Supplemental *ref*. *5*) to map the gains and losses of type III events along the phylogeny. The numbers of gained clusters are in green and lost clusters in red. The total number of type III event clusters for terminal species of the phylogenetic tree is indicated in the parenthesis to the right.
(PDF)

S3 Fig Synonymous/non-synonymous patterns of genomic coding SNPs near A-to-I RNA editing sites in *D*. *melanogaster*. The bar plot displayed the number of genomic SNPs within 500 bp of editing sites from the *D*. *melanogaster* genomic variance database (S2 Text).
(PDF)

S4 Fig The minimum free energy for secondary structures for the sequences flanking editing sites. The box plot distribution of the minimum free energy for secondary structures for the sequences flanking editing sites and randomly selected sequences, calculated using ViennaRNA package (see Methods for details). The *p-valu*e from *Wilcoxon-Mann-Whitney rank sum test* is listed.
(PDF)
