# Supplementary material for: The Landscape of A-to-I RNA Editome Is Shaped by Both Positive and Purifying Selection
Source: PLoS Genet. 2016 Jul 28;12(7):e1006191. doi: 10.1371/journal.pgen.1006191 (PMC4965139; doi:10.1371/journal.pgen.1006191)
Supplement: S3 Text — (DOCX) [file pgen.1006191.s020.docx]

**Supplemental References**

1. Finn RD, Coggill P, Eberhardt RY, Eddy SR, Mistry J, Mitchell AL, et al. The Pfam protein families database: towards a more sustainable future. Nucleic Acids Res. 2016 January 4, 2016;44(D1):D279-D85.

2. Chen D, Berger J, Fellner M, Suzuki T. FLYSNPdb: a high-density SNP database of Drosophila melanogaster. Nucleic Acids Res. 2009;37(Database issue):D567-D70.

3. Nakamura K, Oshima T, Morimoto T, Ikeda S, Yoshikawa H, Shiwa Y, et al. Sequence-specific error profile of Illumina sequencers. Nucleic Acids Res. 2011;39(13):e90-e.

4. Ramaswami G, Zhang R, Piskol R, Keegan LP, Deng P, O'Connell MA, et al. Identifying RNA editing sites using RNA sequencing data alone. Nat Meth. [10.1038/nmeth.2330]. 2013;10(2):128-32.

5. Cohen O, Ashkenazy H, Belinky F, Huchon D, Pupko T. GLOOME: gain loss mapping engine. Bioinformatics. 2010 Nov 15;26(22):2914-5.
