# Supplementary figures and images for: The Landscape of A-to-I RNA Editome Is Shaped by Both Positive and Purifying Selection
Source: PLoS Genet. 2016 Jul 28;12(7):e1006191. doi: 10.1371/journal.pgen.1006191 (PMC4965139; doi:10.1371/journal.pgen.1006191)

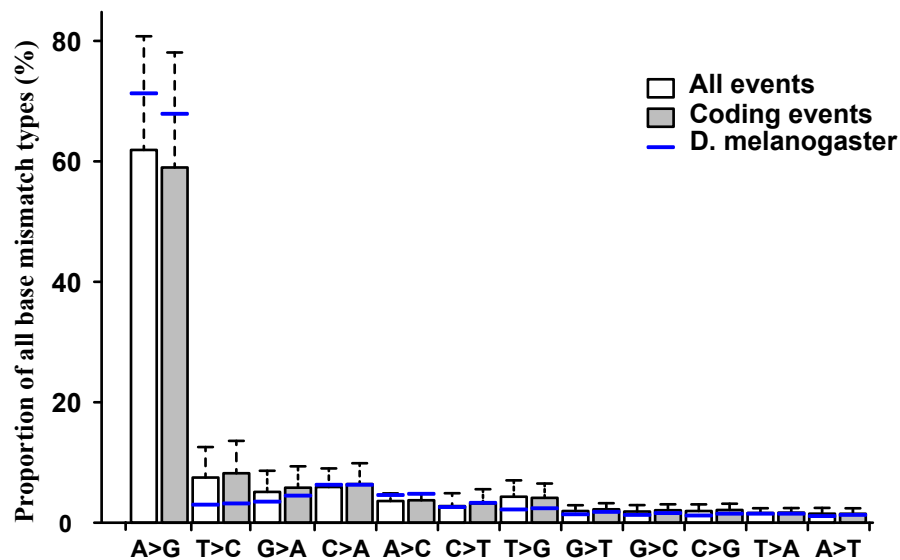

S1 Fig. The proportion of all 12 base change types from 7 *Drosophila* species.

Supplement: S1 Fig — The dash line above each bar represents the standard deviation of corresponding value. The blue line marks the value for D. melanogaster. We applied the same screening method for all base change types (see Methods), and adjusted the parameters according to the basespecific error rates from Illumina sequencing platform (Supplemental ref. 3). Assuming that all non-canonical mismatches were background noise, and the error rates for all 12 base change were equal, the false positive rate for A-to-G change type was estimated to be 5.59% [(38.1%/11)/61.9% = 5.59%] for all sites, and 6.32% [(41.0%/11)/59% = 5.59%] for CDS sites (Supplemental ref. 4). (PDF) [file pgen.1006191.s021.pdf]

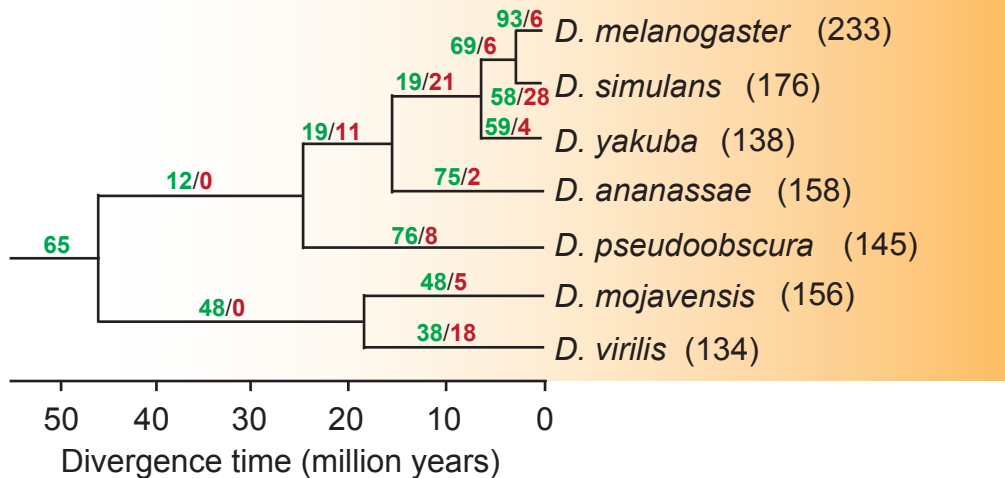

S2 Fig. Events gained and lost in the *Drosophila* lineage.

Supplement: S2 Fig — We used the Gain Loss Mapping Engine (GLOOME) server (Supplemental ref. 5) to map the gains and losses of type III events along the phylogeny. The numbers of gained clusters are in green and lost clusters in red. The total number of type III event clusters for terminal species of the phylogenetic tree is indicated in the parenthesis to the right. (PDF) [file pgen.1006191.s022.pdf]
