# Supplementary material for: The Landscape of A-to-I RNA Editome Is Shaped by Both Positive and Purifying Selection
Source: PLoS Genet. 2016 Jul 28;12(7):e1006191. doi: 10.1371/journal.pgen.1006191 (PMC4965139; doi:10.1371/journal.pgen.1006191)
Supplement: S3 Fig — The bar plot displayed the number of genomic SNPs within 500 bp of editing sites from the D. melanogaster genomic variance database (S2 Text). (PDF) [file pgen.1006191.s023.pdf]

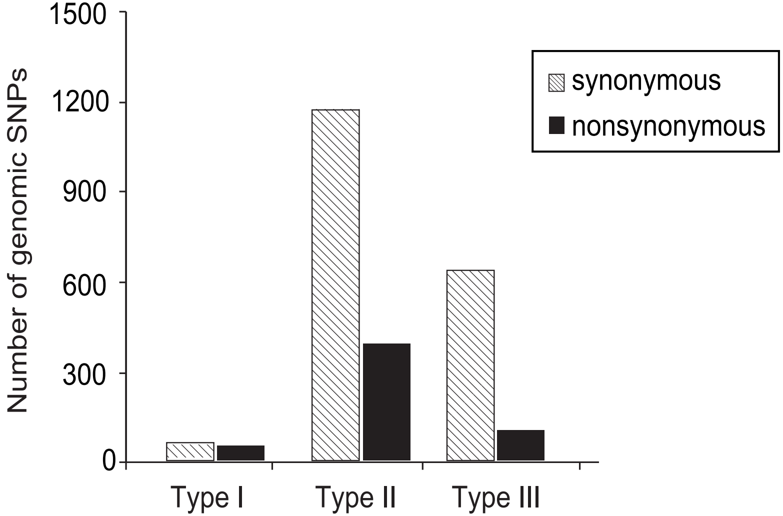

S3 Fig. Synonymous/nonsynonymous paterns of genomic coding SNPs near A-to-I RNA editing sites in D. melanogaster.
