# Supplementary material for: The Landscape of A-to-I RNA Editome Is Shaped by Both Positive and Purifying Selection
Source: PLoS Genet. 2016 Jul 28;12(7):e1006191. doi: 10.1371/journal.pgen.1006191 (PMC4965139; doi:10.1371/journal.pgen.1006191)
Supplement: S4 Fig — The box plot distribution of the minimum free energy for secondary structures for the sequences flanking editing sites and randomly selected sequences, calculated using ViennaRNA package (see Methods for details). The p-value from Wilcoxon-Mann-Whitney rank sum test is listed. (PDF) [file pgen.1006191.s024.pdf]

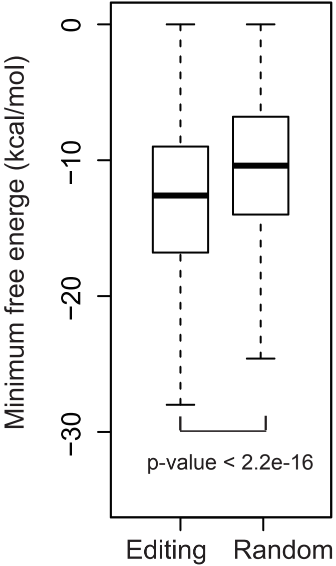

S4 Fig. The minimum free energy for secondary structures for the sequences flanking editing sites.
